# (*R*_C_,*S*_Fe_)-1-[3,5-Bis(trifluoro­meth­yl)phen­yl]-3-{1-[2-(diphenyl­phosphan­yl)ferro­cen­yl]eth­yl}thio­urea (unknown solvate)

**DOI:** 10.1107/S1600536813008453

**Published:** 2013-04-05

**Authors:** Peng-Fei Ma, Xiao-Rui Zhang, Jiang-Wei Ma, Hui Chen, Ru Jiang

**Affiliations:** a Department of Medicinal Chemistry, School of Pharmacy, Fourth Military Medical University, Changle Xilu 169, 710032 Xi-An, People’s Republic of China; b Pharmacy Department of 323 Military Hospital, Jianshe Xilu 6, 710054 Xi-An, People’s Republic of China

## Abstract

In the molecule of the the title compound, [Fe(C_5_H_5_)(C_28_H_22_F_6_N_2_PS)], the absolute configuration is *R*_C_ *,S_Fe_*. The dihedral angle between the trifluoro­methyl-substituted phenyl ring and the thio­urea plane is 41.8 (9)°. The iron atom is bound to the cyclo­penta­dienyl rings in the typical η^5^-manner in a close to eclipsed conformation. The crystal structure features N—H⋯S hydrogen bonds, with the S atom as an acceptor for both N—H groups, forming a layered arrangement parallel to (1-10). The two –CF_3_ groups are each disordered over two positions with refined occupancy rates for the major components of 0.66 (7) and 0.55 (5). The crystal was grown from mixed solvents (*n*-hexane and ethyl acetate). These solvents are disordered in the crystal and the resulting electron density was found to be uninterpretable. The solvent contribution to the structure factors was taken into account by back-Fourier transformation of all density found in the disordered solvent area using the SQUEEZE routine in *PLATON* [Spek (2009 ▶). *Acta Cryst.* D**65**, 148–155]. The formula mass and density do not take account of the solvent.

## Related literature

For an introduction to the Morita–Baylis–Hillman reaction, see: Basavaiah *et al.* (2010 ▶). For the synthesis of (*R_C_,S_Fe_*)-1-(2-(diphenyl­phosphan­yl)ferrocen­yl)ethanamine and structures related to the title compound, see: Chen *et al.* (2006 ▶). For the synthesis of the title compound, see: Sohtome *et al.* (2004 ▶). For refinement details concerning the use of SQUEEZE, see: Spek (2009 ▶).
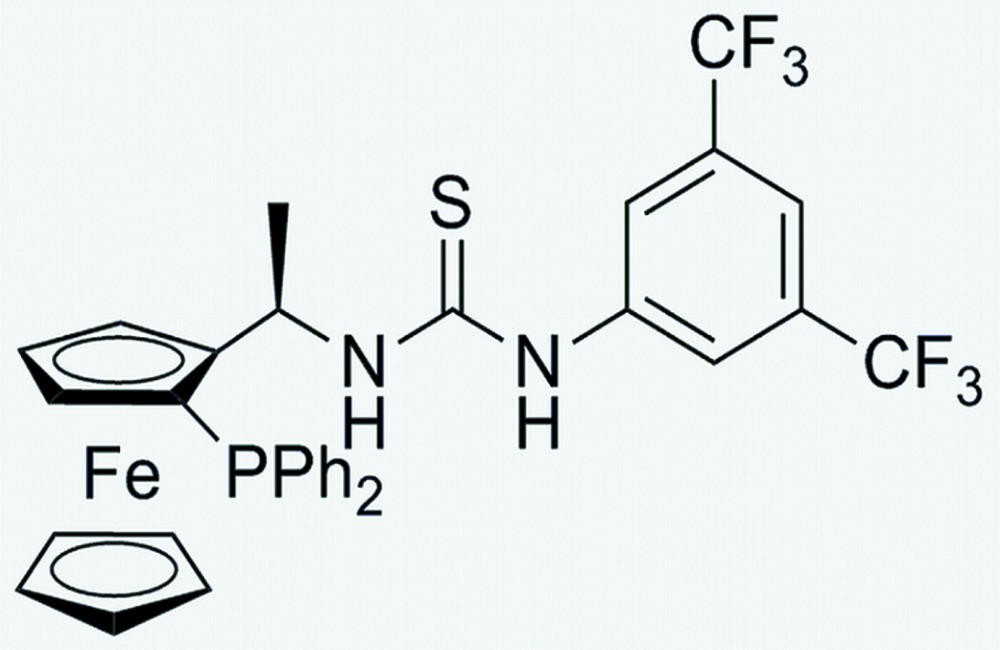


## Experimental

### 

#### Crystal data

[Fe(C_5_H_5_)(C_28_H_22_F_6_N_2_PS)]
*M* *_r_* = 684.45
Tetragonal, 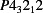

*a* = 20.0898 (16) Å
*c* = 18.012 (2) Å
*V* = 7269.6 (12) Å^3^
*Z* = 8
Mo *K*α radiation
μ = 0.57 mm^−1^
*T* = 296 K
0.37 × 0.31 × 0.25 mm

#### Data collection

Bruker APEXII CCD area-detector diffractometer
Absorption correction: multi-scan (*SADABS*; Bruker, 2008 ▶) *T*_min_ = 0.818, *T*_max_ = 0.869
36567 measured reflections
6472 independent reflections
3487 reflections with *I* > 2σ(*I*)
*R*_int_ = 0.116

#### Refinement

*R*[*F* ^2^ > 2σ(*F* ^2^)] = 0.054
*wR*(*F* ^2^) = 0.119
*S* = 0.95
6472 reflections
455 parameters
H-atom parameters constrained
Δρ_max_ = 0.21 e Å^−3^
Δρ_min_ = −0.19 e Å^−3^
Absolute structure: Flack (1983 ▶), 3680 Friedel pairs
Flack parameter: 0.01 (2)

### 

Data collection: *APEX2* (Bruker, 2010 ▶); cell refinement: *SAINT* (Bruker, 2008 ▶); data reduction: *SAINT*; program(s) used to solve structure: *SHELXS97* (Sheldrick, 2008 ▶); program(s) used to refine structure: *SHELXL97* (Sheldrick, 2008 ▶); molecular graphics: *SHELXTL* (Sheldrick, 2008 ▶); software used to prepare material for publication: *SHELXTL*.

## Supplementary Material

Crystal structure: contains datablock(s) I, global. DOI: 10.1107/S1600536813008453/zl2541sup1.cif

Structure factors: contains datablock(s) I. DOI: 10.1107/S1600536813008453/zl2541Isup2.hkl

Additional supplementary materials:  crystallographic information; 3D view; checkCIF report

## Figures and Tables

**Table 1:** Hydrogen-bond geometry (Å, °)

*D*—H⋯*A*	*D*—H	H⋯*A*	*D*⋯*A*	*D*—H⋯*A*
N1—H1⋯S1^i^	0.86	2.46	3.302 (5)	168
N2—H2⋯S1^i^	0.86	2.64	3.445 (4)	157

Symmetry code: (i) 
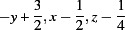
.

**Fig. 1.**
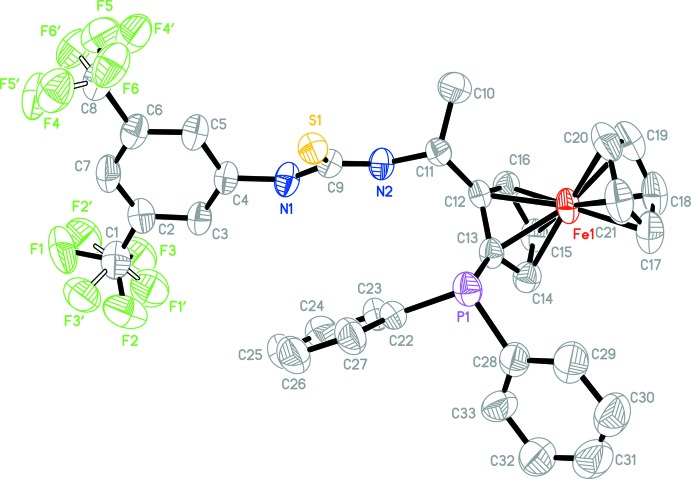
The molecular structure of the title compound, with displacement ellipsoids at the 30% probability level. All H atoms have been omitted for clarity. The disorder of the CF3 groups has been indicated.

**Fig. 2.**
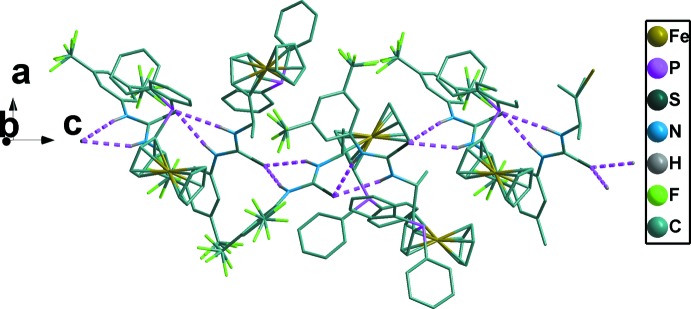
Crystal packing in the title compound where molecules are linked via N–H···S hydrogen bonds (dashed lines). Except for those involved in hydrogen-bonding interactions, H atoms have been omitted for clarity.

## supplementary crystallographic information

### Comment

The compound, synthesized by reaction of 1-isothiocyanato-3,5-bis (trifluoromethyl)benzene with (*R_C_,S_Fe_*)-1-(2-(diphenylphosphanyl)ferrocenyl)ethanamine (Chen *et al.*, 2006), is part of our work towards the synthesis of new phosphanylthiourea ferrocenyl derivatives and their applications in the Morita–Baylis–Hillman (MBH) reaction. The MBH reaction is an atom-economic carbon-carbon bond-forming reaction between the α–position of activated alkenes (alkynes) with carbon electrophiles under the influence of tertiary amines or alkyl (aryl) phosphines (Basavaiah *et al.*, 2010).

The absolute configuration of the title molecule is *R*_C_*,S_Fe_* (Chen *et al.*, 2006). The dihedral angle between the trifluoromethyl substituted phenyl ring C2 → C7 and the plane (S1, C9, N2, N1, H2, H1) comprising the thiourea moiety is 41.8 (9) °. The structure is stabilized by intermolecular N—H···S hydrogen bonds (Fig. 2), with the S atom an acceptor for both N—H groups with an H1···S1 distance of 2.46 Å and an H2···S1 distance of 2.64 Å. The N—H···S angles are 168.4 ° and 165.5 ° (Table 1).

The iron center is bound to the cyclopentadienyl rings in the typical η^5^ manner. The ferrocene group deviates ca. 5 ° from an ideal eclipsed conformation. The angle between the planes of the two cyclopentadienyl rings is 2.8 (9) °.

There are solvent acessible voids in the crystal structure that accomodate solvent molecules in a very disordered way which were corrected for by back-Fourier transformation of all density found in the disordered solvent area using the Squeeze algorithm as implemented in Platon (Spek, 2009). These solvent molecules were not included in the calculation of the overall formula weight, density and absorption coefficient (see refinement section for details).

### Experimental

The title compound was prepared as follows (Sohtome *et al.*, 2004): To a solution of (*R_C_,S_Fe_*)-1-(2-(diphenylphosphanyl)ferrocenyl) ethanamine (2.19 g, 5.30 mmol) in 20 ml THF was added 1-isothiocyanato-3,5-bis (trifluoromethyl)benzene (1.58 g, 5.8 mmol) at room temperature, and the reaction mixture was stirred for 4 h. The solvent was removed under reduced pressure. The residue was purified by column chromatography (silica gel 60–120 mesh, EtOAc/*n*-hexane 1/5) to provide the desired pure title product as a yellow solid (3.44 g, 95%). mp = 404 K; IR (KBr, cm^-1^): ν 3488, 3420, 3235, 2982, 2930, 2028, 1617, 1528, 1381, 1277, 1178, 1135, 886, 748, 698; ^1^H NMR (CDCl_3_, 500 MHz): δ 7.73 (s, 3H), 7.37–7.30 (m, 5H), 7.22 (s, 5H), 7.10 (s, 2H), 5.59–5.58 (m, 1H), 4.51 (s, 1H), 4.33 (s, 1H), 3.96 (s, 5H), 3.78 (s, 1H), 1.46 (s, 3H); ^13^C NMR (CDCl_3_, 126 MHz): δ 138.92, 135.82, 134.84, 134.67, 132.34, 129.53, 128.30, 128.21 (d, *J* = 7.5 Hz), 124.83, 124.01, 121.83, 119.45, 95.04 (d, *J* = 24.6 Hz), 73.21, 72.13, 71.27, 69.85, 69.70, 65.86, 22.26, 15.28; ^31^P NMR (CDCl_3_, 202 MHz): δ -24.79; HRMS calcd for C_33_H_27_FeN_2_PS([*M*+1]^+^): 684.0886, found: 685.0931.

Single crystals were obtained from a solution of the title compound in a mixture of *n*-hexane and EtOAc.

### Refinement

All H atoms were placed in idealized positions and allowed to ride on the respective parent atom with C—H distances of 0.98 Å (ferrocenyl), 0.93 Å (aromatic), 0.96 Å (CH_3_), or 0.98 Å (CH) and N—H distance of 0.86 Å, and with *U*_iso_(H) = 1.5 *U*_eq_(C) for methyl H atoms, with *U*_iso_(H) = 1.2 *U*_eq_(C) for aromatic and methylene H atoms.

In the crystal molecule, the two CF_3_ groups are both disordered over two orientations. The refined occupancy rate for the major moiety of group (F1, F2, F3) is 0.55 (5) [0.66 (7) for group (F4, F5, F6)].

Some residual electron densities were difficult to model, therefore the SQUEEZE function of *PLATON* (Spek, 2009) was used to eliminate the contribution of the electron density in the solvent region from the intensity data, and the solvent-free model was employed for the final refinement. There are two cavities of 652 Å^3^ per unit cell. *PLATON* estimated that each cavity contains 74 electrons. PLATON estimated that each single crystal molecular contains 20 residual electrons. Because single crystals were obtained from a solution of the title compound in a mixture of n-hexane and EtOAc, so we could not be sure which solvent it was. It may be n-hexane [CH_3_(CH_2_)3CH_3_], EtOAc (CH_3_COOCH_2_CH_3_) or water (H_2_O). The solvent is therefore not given in the formula, scheme, Mr etc. .

### Crystal data

**Table:**

[Fe(C_5_H_5_)(C_28_H_22_F_6_N_2_PS)]	*D*_x_ = 1.251 Mg m^−^^3^
*M**_r_* = 684.45	Mo *K*α radiation, λ = 0.71073 Å
Tetragonal, *P*4_3_2_1_2	Cell parameters from 3087 reflections
Hall symbol: P 4nw 2abw	θ = 2.5–15.4°
*a* = 20.0898 (16) Å	µ = 0.57 mm^−^^1^
*c* = 18.012 (2) Å	*T* = 296 K
*V* = 7269.6 (12) Å^3^	Column, yellow
*Z* = 8	0.37 × 0.31 × 0.25 mm
*F*(000) = 2800	

### Data collection

**Table:**

Bruker APEXII CCD area-detector diffractometer	6472 independent reflections
Radiation source: fine-focus sealed tube	3487 reflections with *I* > 2σ(*I*)
Graphite monochromator	*R*_int_ = 0.116
phi and ω scans	θ_max_ = 25.1°, θ_min_ = 1.8°
Absorption correction: multi-scan (*SADABS*; Bruker, 2008)	*h* = −23→21
*T*_min_ = 0.818, *T*_max_ = 0.869	*k* = −23→22
36567 measured reflections	*l* = −21→21

### Refinement

**Table:**

Refinement on *F*^2^	Hydrogen site location: inferred from neighbouring sites
Least-squares matrix: full	H-atom parameters constrained
*R*[*F*^2^ > 2σ(*F*^2^)] = 0.054	*w* = 1/[σ^2^(*F*_o_^2^) + (0.0469*P*)^2^] where *P* = (*F*_o_^2^ + 2*F*_c_^2^)/3
*wR*(*F*^2^) = 0.119	(Δ/σ)_max_ = 0.001
*S* = 0.95	Δρ_max_ = 0.21 e Å^−^^3^
6472 reflections	Δρ_min_ = −0.19 e Å^−^^3^
455 parameters	Extinction correction: *SHELXL97* (Sheldrick, 2008), Fc^*^=kFc[1+0.001xFc^2^λ^3^/sin(2θ)]^-1/4^
0 restraints	Extinction coefficient: 0.00072 (19)
Primary atom site location: structure-invariant direct methods	Absolute structure: Flack (1983), 3680 Friedel pairs
Secondary atom site location: difference Fourier map	Flack parameter: 0.01 (2)

### Special details

**Table:**

Geometry. All e.s.d.'s (except the e.s.d. in the dihedral angle between two l.s. planes) are estimated using the full covariance matrix. The cell e.s.d.'s are taken into account individually in the estimation of e.s.d.'s in distances, angles and torsion angles; correlations between e.s.d.'s in cell parameters are only used when they are defined by crystal symmetry. An approximate (isotropic) treatment of cell e.s.d.'s is used for estimating e.s.d.'s involving l.s. planes.
Refinement. Refinement of *F*^2^ against ALL reflections. The weighted *R*-factor *wR* and goodness of fit *S* are based on *F*^2^, conventional *R*-factors *R* are based on *F*, with *F* set to zero for negative *F*^2^. The threshold expression of *F*^2^ > σ(*F*^2^) is used only for calculating *R*-factors(gt) *etc*. and is not relevant to the choice of reflections for refinement. *R*-factors based on *F*^2^ are statistically about twice as large as those based on *F*, and *R*- factors based on ALL data will be even larger.

### Fractional atomic coordinates and isotropic or equivalent isotropic displacement parameters (Å^2^)

**Table:**

	*x*	*y*	*z*	*U*_iso_*/*U*_eq_	Occ. (<1)
Fe1	1.03592 (4)	0.27046 (4)	0.90856 (4)	0.0791 (3)	
P1	0.87204 (7)	0.31616 (7)	0.91033 (8)	0.0779 (4)	
S1	0.88996 (6)	0.53723 (7)	0.86099 (6)	0.0725 (4)	
N1	0.9005 (2)	0.5092 (2)	0.7164 (2)	0.0824 (13)	
H1	0.9129	0.4805	0.6838	0.099*	
N2	0.9655 (2)	0.44743 (19)	0.7912 (2)	0.0723 (11)	
H2	0.9774	0.4304	0.7495	0.087*	
F1	0.7195 (16)	0.6313 (10)	0.4957 (19)	0.169 (12)	0.55 (5)
F2	0.6984 (12)	0.533 (2)	0.5454 (8)	0.165 (12)	0.55 (5)
F3	0.7835 (11)	0.5518 (18)	0.4735 (9)	0.141 (9)	0.55 (5)
F4	0.7825 (10)	0.7839 (8)	0.683 (2)	0.136 (5)	0.66 (7)
F5	0.8914 (15)	0.7744 (15)	0.678 (2)	0.129 (7)	0.66 (7)
F6	0.835 (2)	0.7507 (6)	0.7759 (6)	0.127 (9)	0.66 (7)
F1'	0.753 (2)	0.5192 (16)	0.500 (2)	0.166 (16)	0.45 (5)
F2'	0.755 (2)	0.6185 (17)	0.4681 (12)	0.154 (12)	0.45 (5)
F3'	0.6802 (10)	0.586 (2)	0.5446 (9)	0.143 (11)	0.45 (5)
F4'	0.883 (4)	0.7539 (10)	0.770 (2)	0.131 (12)	0.34 (7)
F5'	0.789 (2)	0.774 (2)	0.726 (4)	0.134 (16)	0.34 (7)
F6'	0.864 (3)	0.786 (2)	0.659 (2)	0.117 (12)	0.34 (7)
C1	0.7441 (6)	0.5779 (6)	0.5286 (6)	0.127 (3)	
C2	0.7878 (3)	0.5970 (4)	0.5932 (3)	0.0953 (17)	
C3	0.8220 (3)	0.5467 (3)	0.6282 (3)	0.0840 (16)	
H3	0.8185	0.5030	0.6114	0.101*	
C4	0.8613 (3)	0.5617 (3)	0.6880 (3)	0.0769 (15)	
C5	0.8671 (3)	0.6269 (3)	0.7140 (3)	0.0844 (15)	
H5	0.8947	0.6368	0.7539	0.101*	
C6	0.8310 (3)	0.6761 (3)	0.6793 (3)	0.0855 (17)	
C7	0.7920 (3)	0.6615 (4)	0.6185 (4)	0.0983 (19)	
H7	0.7685	0.6952	0.5946	0.118*	
C8	0.8385 (6)	0.7456 (4)	0.7049 (5)	0.107 (2)	
C9	0.9223 (3)	0.4961 (3)	0.7875 (3)	0.0687 (13)	
C10	1.0638 (3)	0.4510 (3)	0.8698 (4)	0.114 (2)	
H10A	1.0577	0.4960	0.8862	0.171*	
H10B	1.0885	0.4508	0.8242	0.171*	
H10C	1.0878	0.4264	0.9068	0.171*	
C11	0.9958 (2)	0.4186 (2)	0.8571 (3)	0.0687 (13)	
H11	0.9673	0.4275	0.9001	0.082*	
C12	0.9997 (2)	0.3449 (2)	0.8450 (2)	0.0636 (12)	
C13	0.9480 (2)	0.2979 (3)	0.8596 (2)	0.0663 (13)	
C14	0.9697 (3)	0.2346 (3)	0.8330 (3)	0.0825 (15)	
H14	0.9451	0.1927	0.8367	0.099*	
C15	1.0332 (3)	0.2433 (3)	0.7997 (3)	0.0829 (16)	
H15	1.0601	0.2083	0.7765	0.099*	
C16	1.0515 (3)	0.3111 (3)	0.8070 (3)	0.0776 (15)	
H16	1.0932	0.3311	0.7896	0.093*	
C17	1.0393 (4)	0.2044 (4)	0.9946 (4)	0.115 (2)	
H17	1.0116	0.1646	1.0001	0.138*	
C18	1.1014 (4)	0.2080 (5)	0.9573 (4)	0.121 (2)	
H18	1.1246	0.1709	0.9332	0.146*	
C19	1.1240 (4)	0.2719 (5)	0.9628 (4)	0.118 (2)	
H19	1.1661	0.2886	0.9425	0.141*	
C20	1.0774 (4)	0.3099 (4)	1.0026 (4)	0.106 (2)	
H20	1.0811	0.3574	1.0146	0.127*	
C21	1.0246 (4)	0.2680 (5)	1.0218 (3)	0.108 (2)	
H21	0.9846	0.2807	1.0495	0.130*	
C22	0.8187 (3)	0.3452 (3)	0.8353 (3)	0.0748 (14)	
C23	0.8331 (3)	0.3392 (3)	0.7626 (4)	0.0925 (18)	
H23	0.8721	0.3177	0.7487	0.111*	
C24	0.7910 (4)	0.3642 (4)	0.7074 (4)	0.113 (2)	
H24	0.8013	0.3596	0.6573	0.135*	
C25	0.7343 (4)	0.3957 (4)	0.7299 (5)	0.115 (2)	
H25	0.7059	0.4133	0.6941	0.139*	
C26	0.7185 (3)	0.4020 (4)	0.7996 (5)	0.120 (2)	
H26	0.6791	0.4231	0.8132	0.144*	
C27	0.7606 (3)	0.3772 (3)	0.8526 (4)	0.1024 (18)	
H27	0.7492	0.3823	0.9023	0.123*	
C28	0.8417 (3)	0.2309 (3)	0.9269 (4)	0.0954 (19)	
C29	0.8552 (3)	0.2030 (4)	0.9948 (4)	0.124 (2)	
H29	0.8762	0.2281	1.0313	0.149*	
C30	0.8373 (4)	0.1364 (5)	1.0092 (6)	0.144 (3)	
H30	0.8475	0.1167	1.0546	0.173*	
C31	0.8057 (5)	0.1022 (5)	0.9568 (7)	0.148 (4)	
H31	0.7949	0.0579	0.9660	0.178*	
C32	0.7886 (4)	0.1290 (5)	0.8910 (6)	0.147 (3)	
H32	0.7641	0.1048	0.8565	0.177*	
C33	0.8088 (3)	0.1945 (4)	0.8757 (4)	0.112 (2)	
H33	0.7993	0.2131	0.8296	0.134*	

### Atomic displacement parameters (Å^2^)

**Table:**

	*U*^11^	*U*^22^	*U*^33^	*U*^12^	*U*^13^	*U*^23^
Fe1	0.0709 (5)	0.0911 (6)	0.0755 (5)	0.0309 (4)	0.0026 (4)	0.0142 (4)
P1	0.0686 (9)	0.0906 (11)	0.0745 (8)	0.0174 (8)	0.0099 (8)	0.0051 (8)
S1	0.0756 (9)	0.0757 (9)	0.0663 (7)	0.0083 (7)	−0.0027 (7)	−0.0090 (7)
N1	0.109 (3)	0.067 (3)	0.071 (3)	0.025 (3)	−0.001 (3)	0.000 (2)
N2	0.079 (3)	0.071 (3)	0.067 (3)	0.017 (2)	0.004 (2)	0.003 (2)
F1	0.15 (2)	0.190 (13)	0.16 (2)	0.070 (14)	−0.058 (17)	0.021 (14)
F2	0.117 (12)	0.20 (3)	0.175 (10)	−0.037 (17)	−0.021 (9)	−0.004 (12)
F3	0.130 (11)	0.18 (2)	0.109 (8)	−0.002 (11)	−0.027 (7)	−0.005 (9)
F4	0.168 (10)	0.092 (5)	0.150 (14)	0.053 (5)	−0.010 (10)	0.022 (8)
F5	0.127 (12)	0.101 (11)	0.158 (19)	−0.013 (8)	0.028 (10)	−0.013 (10)
F6	0.19 (3)	0.090 (5)	0.097 (6)	0.021 (8)	0.012 (9)	−0.006 (4)
F1'	0.18 (3)	0.165 (19)	0.16 (2)	−0.008 (18)	−0.06 (2)	−0.016 (16)
F2'	0.15 (2)	0.18 (3)	0.131 (12)	0.003 (19)	−0.026 (12)	0.004 (12)
F3'	0.101 (11)	0.16 (3)	0.164 (11)	−0.012 (11)	−0.023 (8)	0.024 (12)
F4'	0.16 (4)	0.083 (9)	0.153 (18)	0.006 (12)	−0.011 (17)	0.001 (8)
F5'	0.14 (2)	0.107 (17)	0.16 (3)	0.021 (13)	0.04 (2)	−0.01 (2)
F6'	0.17 (4)	0.082 (11)	0.104 (13)	−0.005 (18)	0.027 (19)	0.025 (9)
C1	0.135 (10)	0.114 (9)	0.134 (9)	0.025 (8)	−0.011 (8)	0.013 (7)
C2	0.098 (5)	0.100 (5)	0.088 (4)	0.022 (4)	−0.006 (4)	0.006 (4)
C3	0.094 (4)	0.079 (4)	0.079 (4)	0.018 (3)	−0.001 (3)	0.018 (3)
C4	0.091 (4)	0.070 (4)	0.070 (3)	0.019 (3)	0.001 (3)	0.012 (3)
C5	0.101 (4)	0.074 (4)	0.078 (4)	0.022 (3)	0.011 (3)	0.008 (3)
C6	0.100 (5)	0.071 (4)	0.086 (4)	0.017 (4)	0.022 (3)	0.009 (3)
C7	0.096 (5)	0.097 (5)	0.102 (5)	0.031 (4)	0.003 (4)	0.033 (4)
C8	0.125 (8)	0.088 (7)	0.110 (7)	0.023 (6)	0.023 (6)	0.014 (5)
C9	0.074 (4)	0.063 (3)	0.069 (3)	0.003 (3)	0.001 (3)	0.002 (3)
C10	0.096 (5)	0.092 (5)	0.155 (6)	0.001 (4)	−0.028 (4)	−0.011 (4)
C11	0.069 (3)	0.073 (4)	0.064 (3)	0.002 (3)	−0.010 (3)	0.003 (3)
C12	0.056 (3)	0.071 (3)	0.064 (3)	0.013 (3)	−0.007 (3)	0.005 (3)
C13	0.059 (3)	0.077 (4)	0.062 (3)	0.015 (3)	0.009 (2)	0.011 (3)
C14	0.083 (4)	0.082 (4)	0.083 (4)	0.009 (3)	0.006 (3)	0.006 (3)
C15	0.088 (4)	0.085 (4)	0.075 (3)	0.023 (3)	0.013 (3)	0.000 (3)
C16	0.072 (4)	0.079 (4)	0.082 (4)	0.006 (3)	0.006 (3)	0.010 (3)
C17	0.119 (7)	0.125 (7)	0.102 (5)	0.031 (5)	−0.004 (5)	0.037 (4)
C18	0.113 (6)	0.129 (7)	0.122 (6)	0.055 (5)	−0.014 (5)	0.028 (5)
C19	0.092 (5)	0.155 (7)	0.105 (5)	0.023 (6)	−0.024 (4)	0.009 (6)
C20	0.104 (5)	0.126 (6)	0.087 (4)	0.025 (5)	−0.035 (4)	−0.009 (4)
C21	0.112 (6)	0.131 (6)	0.080 (4)	0.034 (5)	−0.011 (4)	0.019 (4)
C22	0.061 (3)	0.071 (3)	0.092 (4)	0.002 (3)	−0.006 (3)	0.007 (3)
C23	0.081 (4)	0.095 (4)	0.102 (5)	0.013 (3)	−0.016 (4)	0.017 (4)
C24	0.105 (6)	0.119 (6)	0.114 (5)	−0.014 (5)	−0.017 (5)	0.020 (5)
C25	0.091 (5)	0.116 (6)	0.140 (7)	0.008 (5)	−0.031 (5)	0.023 (5)
C26	0.090 (5)	0.114 (6)	0.156 (7)	0.021 (4)	−0.021 (6)	−0.009 (6)
C27	0.080 (4)	0.105 (5)	0.121 (5)	0.023 (4)	−0.013 (4)	−0.006 (4)
C28	0.068 (4)	0.105 (5)	0.114 (5)	0.012 (4)	0.017 (4)	0.032 (5)
C29	0.096 (5)	0.135 (7)	0.142 (7)	0.018 (5)	0.024 (5)	0.043 (5)
C30	0.119 (7)	0.145 (9)	0.168 (9)	0.018 (6)	0.032 (6)	0.063 (7)
C31	0.108 (7)	0.136 (8)	0.201 (11)	0.005 (6)	0.041 (7)	0.047 (8)
C32	0.117 (6)	0.135 (8)	0.190 (9)	0.002 (6)	0.007 (6)	0.041 (7)
C33	0.096 (5)	0.099 (5)	0.140 (6)	−0.017 (4)	0.016 (5)	0.023 (5)

### Geometric parameters (Å, º)

**Table:**

Fe1—C18	2.018 (6)	C10—H10B	0.9600
Fe1—C12	2.019 (4)	C10—H10C	0.9600
Fe1—C19	2.021 (6)	C11—C12	1.497 (6)
Fe1—C16	2.028 (5)	C11—H11	0.9800
Fe1—C14	2.034 (5)	C12—C16	1.419 (6)
Fe1—C15	2.036 (5)	C12—C13	1.428 (6)
Fe1—C17	2.040 (6)	C13—C14	1.426 (6)
Fe1—C20	2.048 (6)	C14—C15	1.420 (7)
Fe1—C13	2.049 (4)	C14—H14	0.9800
Fe1—C21	2.053 (6)	C15—C16	1.416 (7)
P1—C13	1.817 (5)	C15—H15	0.9800
P1—C22	1.820 (5)	C16—H16	0.9800
P1—C28	1.843 (7)	C17—C21	1.399 (8)
S1—C9	1.690 (5)	C17—C18	1.419 (9)
N1—C9	1.378 (6)	C17—H17	0.9800
N1—C4	1.413 (6)	C18—C19	1.366 (9)
N1—H1	0.8600	C18—H18	0.9800
N2—C9	1.309 (5)	C19—C20	1.405 (9)
N2—C11	1.455 (5)	C19—H19	0.9800
N2—H2	0.8600	C20—C21	1.398 (8)
F1—C1	1.32 (2)	C20—H20	0.9800
F2—C1	1.32 (2)	C21—H21	0.9800
F3—C1	1.37 (2)	C22—C23	1.346 (7)
F4—C8	1.42 (2)	C22—C27	1.370 (7)
F5—C8	1.30 (2)	C23—C24	1.399 (7)
F6—C8	1.286 (13)	C23—H23	0.9300
F1'—C1	1.30 (2)	C24—C25	1.365 (9)
F2'—C1	1.38 (2)	C24—H24	0.9300
F3'—C1	1.33 (3)	C25—C26	1.302 (8)
F4'—C8	1.49 (3)	C25—H25	0.9300
F5'—C8	1.22 (4)	C26—C27	1.369 (8)
F6'—C8	1.27 (4)	C26—H26	0.9300
C1—C2	1.508 (10)	C27—H27	0.9300
C2—C3	1.375 (7)	C28—C33	1.349 (8)
C2—C7	1.377 (8)	C28—C29	1.373 (8)
C3—C4	1.369 (7)	C29—C30	1.410 (10)
C3—H3	0.9300	C29—H29	0.9300
C4—C5	1.396 (7)	C30—C31	1.330 (10)
C5—C6	1.377 (7)	C30—H30	0.9300
C5—H5	0.9300	C31—C32	1.348 (10)
C6—C7	1.378 (7)	C31—H31	0.9300
C6—C8	1.478 (9)	C32—C33	1.403 (9)
C7—H7	0.9300	C32—H32	0.9300
C10—C11	1.529 (7)	C33—H33	0.9300
C10—H10A	0.9600		
			
C18—Fe1—C12	160.3 (3)	N2—C9—S1	125.4 (4)
C18—Fe1—C19	39.5 (3)	N1—C9—S1	120.8 (4)
C12—Fe1—C19	125.3 (3)	C11—C10—H10A	109.5
C18—Fe1—C16	122.8 (3)	C11—C10—H10B	109.5
C12—Fe1—C16	41.04 (18)	H10A—C10—H10B	109.5
C19—Fe1—C16	107.2 (3)	C11—C10—H10C	109.5
C18—Fe1—C14	119.8 (3)	H10A—C10—H10C	109.5
C12—Fe1—C14	69.4 (2)	H10B—C10—H10C	109.5
C19—Fe1—C14	154.3 (3)	N2—C11—C12	107.3 (4)
C16—Fe1—C14	68.9 (2)	N2—C11—C10	109.0 (4)
C18—Fe1—C15	105.6 (3)	C12—C11—C10	113.3 (4)
C12—Fe1—C15	69.09 (19)	N2—C11—H11	109.0
C19—Fe1—C15	119.5 (3)	C12—C11—H11	109.0
C16—Fe1—C15	40.79 (18)	C10—C11—H11	109.0
C14—Fe1—C15	40.85 (19)	C16—C12—C13	107.7 (4)
C18—Fe1—C17	40.9 (2)	C16—C12—C11	125.6 (5)
C12—Fe1—C17	157.5 (3)	C13—C12—C11	126.1 (4)
C19—Fe1—C17	67.3 (3)	C16—C12—Fe1	69.8 (3)
C16—Fe1—C17	160.3 (3)	C13—C12—Fe1	70.6 (3)
C14—Fe1—C17	107.4 (3)	C11—C12—Fe1	132.0 (3)
C15—Fe1—C17	123.9 (3)	C14—C13—C12	107.8 (4)
C18—Fe1—C20	67.4 (3)	C14—C13—P1	127.3 (4)
C12—Fe1—C20	109.2 (3)	C12—C13—P1	124.7 (4)
C19—Fe1—C20	40.4 (2)	C14—C13—Fe1	69.0 (3)
C16—Fe1—C20	121.9 (3)	C12—C13—Fe1	68.3 (3)
C14—Fe1—C20	163.0 (3)	P1—C13—Fe1	124.2 (2)
C15—Fe1—C20	155.7 (3)	C15—C14—C13	107.9 (5)
C17—Fe1—C20	67.1 (3)	C15—C14—Fe1	69.7 (3)
C18—Fe1—C13	156.3 (4)	C13—C14—Fe1	70.1 (3)
C12—Fe1—C13	41.10 (17)	C15—C14—H14	126.1
C19—Fe1—C13	163.4 (3)	C13—C14—H14	126.1
C16—Fe1—C13	68.64 (19)	Fe1—C14—H14	126.1
C14—Fe1—C13	40.87 (18)	C16—C15—C14	108.1 (5)
C15—Fe1—C13	68.5 (2)	C16—C15—Fe1	69.3 (3)
C17—Fe1—C13	122.0 (3)	C14—C15—Fe1	69.5 (3)
C20—Fe1—C13	127.1 (3)	C16—C15—H15	125.9
C18—Fe1—C21	68.0 (3)	C14—C15—H15	125.9
C12—Fe1—C21	122.8 (3)	Fe1—C15—H15	125.9
C19—Fe1—C21	67.5 (3)	C15—C16—C12	108.4 (5)
C16—Fe1—C21	157.4 (3)	C15—C16—Fe1	69.9 (3)
C14—Fe1—C21	125.7 (3)	C12—C16—Fe1	69.2 (3)
C15—Fe1—C21	161.3 (3)	C15—C16—H16	125.8
C17—Fe1—C21	40.0 (2)	C12—C16—H16	125.8
C20—Fe1—C21	39.9 (2)	Fe1—C16—H16	125.8
C13—Fe1—C21	109.8 (2)	C21—C17—C18	107.8 (7)
C13—P1—C22	100.7 (2)	C21—C17—Fe1	70.5 (4)
C13—P1—C28	99.9 (3)	C18—C17—Fe1	68.7 (4)
C22—P1—C28	102.9 (3)	C21—C17—H17	126.1
C9—N1—C4	131.0 (4)	C18—C17—H17	126.1
C9—N1—H1	114.5	Fe1—C17—H17	126.1
C4—N1—H1	114.5	C19—C18—C17	107.8 (7)
C9—N2—C11	128.1 (4)	C19—C18—Fe1	70.3 (4)
C9—N2—H2	116.0	C17—C18—Fe1	70.4 (4)
C11—N2—H2	116.0	C19—C18—H18	126.1
F1'—C1—F2	64.6 (18)	C17—C18—H18	126.1
F1'—C1—F1	127.5 (14)	Fe1—C18—H18	126.1
F2—C1—F1	113.3 (16)	C18—C19—C20	109.1 (7)
F1'—C1—F3'	109.3 (19)	C18—C19—Fe1	70.1 (4)
F2—C1—F3'	50.1 (11)	C20—C19—Fe1	70.8 (4)
F1—C1—F3'	68.7 (11)	C18—C19—H19	125.4
F2—C1—F3	107.8 (15)	C20—C19—H19	125.4
F1—C1—F3	101.6 (16)	Fe1—C19—H19	125.4
F3'—C1—F3	139.6 (12)	C21—C20—C19	107.6 (7)
F1'—C1—F2'	101.4 (19)	C21—C20—Fe1	70.2 (4)
F2—C1—F2'	134.3 (13)	C19—C20—Fe1	68.8 (4)
F3'—C1—F2'	105.1 (15)	C21—C20—H20	126.2
F3—C1—F2'	63.8 (14)	C19—C20—H20	126.2
F1'—C1—C2	117.2 (11)	Fe1—C20—H20	126.2
F2—C1—C2	113.7 (12)	C20—C21—C17	107.7 (7)
F1—C1—C2	111.0 (12)	C20—C21—Fe1	69.9 (4)
F3'—C1—C2	111.5 (13)	C17—C21—Fe1	69.5 (4)
F3—C1—C2	108.6 (11)	C20—C21—H21	126.2
F2'—C1—C2	111.3 (13)	C17—C21—H21	126.2
C3—C2—C7	120.7 (6)	Fe1—C21—H21	126.2
C3—C2—C1	117.2 (7)	C23—C22—C27	116.5 (5)
C7—C2—C1	122.0 (7)	C23—C22—P1	124.6 (5)
C4—C3—C2	119.1 (6)	C27—C22—P1	118.9 (5)
C4—C3—H3	120.4	C22—C23—C24	122.0 (6)
C2—C3—H3	120.4	C22—C23—H23	119.0
C3—C4—C5	121.2 (5)	C24—C23—H23	119.0
C3—C4—N1	116.2 (5)	C25—C24—C23	117.4 (7)
C5—C4—N1	122.2 (5)	C25—C24—H24	121.3
C6—C5—C4	118.6 (5)	C23—C24—H24	121.3
C6—C5—H5	120.7	C26—C25—C24	122.4 (7)
C4—C5—H5	120.7	C26—C25—H25	118.8
C5—C6—C7	120.5 (6)	C24—C25—H25	118.8
C5—C6—C8	118.9 (7)	C25—C26—C27	119.1 (7)
C7—C6—C8	120.5 (7)	C25—C26—H26	120.5
C2—C7—C6	119.8 (6)	C27—C26—H26	120.5
C2—C7—H7	120.1	C26—C27—C22	122.7 (6)
C6—C7—H7	120.1	C26—C27—H27	118.7
F5'—C8—F6'	104 (2)	C22—C27—H27	118.7
F5'—C8—F6	67 (2)	C33—C28—C29	119.0 (7)
F6'—C8—F6	128 (2)	C33—C28—P1	123.7 (6)
F5'—C8—F5	125.2 (19)	C29—C28—P1	117.2 (7)
F6—C8—F5	112.1 (16)	C28—C29—C30	120.1 (8)
F6'—C8—F4	78 (2)	C28—C29—H29	120.0
F6—C8—F4	101.0 (12)	C30—C29—H29	120.0
F5—C8—F4	107.7 (14)	C31—C30—C29	118.8 (10)
F5'—C8—C6	117.4 (17)	C31—C30—H30	120.6
F6'—C8—C6	116 (2)	C29—C30—H30	120.6
F6—C8—C6	112.4 (9)	C30—C31—C32	122.7 (11)
F5—C8—C6	112.7 (14)	C30—C31—H31	118.7
F4—C8—C6	110.3 (11)	C32—C31—H31	118.7
F5'—C8—F4'	101.5 (18)	C31—C32—C33	118.3 (9)
F6'—C8—F4'	102 (2)	C31—C32—H32	120.9
F5—C8—F4'	75.8 (18)	C33—C32—H32	120.9
F4—C8—F4'	129.2 (14)	C28—C33—C32	121.1 (8)
C6—C8—F4'	114.5 (11)	C28—C33—H33	119.5
N2—C9—N1	113.6 (4)	C32—C33—H33	119.5
			
F1'—C1—C2—C3	−15 (4)	C16—Fe1—C15—C14	−119.8 (4)
F2—C1—C2—C3	57 (3)	C17—Fe1—C15—C14	76.9 (4)
F1—C1—C2—C3	−174 (2)	C20—Fe1—C15—C14	−172.7 (6)
F3'—C1—C2—C3	112 (2)	C13—Fe1—C15—C14	−38.0 (3)
F3—C1—C2—C3	−63 (2)	C21—Fe1—C15—C14	50.5 (9)
F2'—C1—C2—C3	−131 (2)	C14—C15—C16—C12	0.2 (5)
F1'—C1—C2—C7	167 (3)	Fe1—C15—C16—C12	−58.6 (3)
F2—C1—C2—C7	−121 (2)	C14—C15—C16—Fe1	58.8 (3)
F1—C1—C2—C7	9 (3)	C13—C12—C16—C15	−1.6 (5)
F3'—C1—C2—C7	−66 (2)	C11—C12—C16—C15	−173.1 (4)
F3—C1—C2—C7	119.4 (19)	Fe1—C12—C16—C15	59.1 (3)
F2'—C1—C2—C7	51 (3)	C13—C12—C16—Fe1	−60.7 (3)
C7—C2—C3—C4	−1.0 (9)	C11—C12—C16—Fe1	127.8 (5)
C1—C2—C3—C4	−178.7 (8)	C18—Fe1—C16—C15	75.1 (5)
C2—C3—C4—C5	0.1 (8)	C12—Fe1—C16—C15	−119.9 (4)
C2—C3—C4—N1	−173.4 (5)	C19—Fe1—C16—C15	115.5 (4)
C9—N1—C4—C3	−149.3 (5)	C14—Fe1—C16—C15	−37.5 (3)
C9—N1—C4—C5	37.2 (8)	C17—Fe1—C16—C15	45.2 (9)
C3—C4—C5—C6	1.6 (8)	C20—Fe1—C16—C15	157.3 (4)
N1—C4—C5—C6	174.7 (5)	C13—Fe1—C16—C15	−81.5 (3)
C4—C5—C6—C7	−2.3 (8)	C21—Fe1—C16—C15	−171.9 (6)
C4—C5—C6—C8	−178.0 (6)	C18—Fe1—C16—C12	−164.9 (4)
C3—C2—C7—C6	0.3 (9)	C19—Fe1—C16—C12	−124.5 (4)
C1—C2—C7—C6	177.9 (8)	C14—Fe1—C16—C12	82.5 (3)
C5—C6—C7—C2	1.4 (9)	C15—Fe1—C16—C12	119.9 (4)
C8—C6—C7—C2	177.0 (7)	C17—Fe1—C16—C12	165.2 (7)
C5—C6—C8—F5'	−122 (5)	C20—Fe1—C16—C12	−82.8 (4)
C7—C6—C8—F5'	62 (5)	C13—Fe1—C16—C12	38.5 (3)
C5—C6—C8—F6'	115 (4)	C21—Fe1—C16—C12	−52.0 (7)
C7—C6—C8—F6'	−61 (4)	C18—Fe1—C17—C21	119.1 (7)
C5—C6—C8—F6	−47 (2)	C12—Fe1—C17—C21	−47.2 (9)
C7—C6—C8—F6	137 (2)	C19—Fe1—C17—C21	81.6 (5)
C5—C6—C8—F5	81 (2)	C16—Fe1—C17—C21	158.9 (7)
C7—C6—C8—F5	−95 (2)	C14—Fe1—C17—C21	−125.3 (5)
C5—C6—C8—F4	−158.7 (16)	C15—Fe1—C17—C21	−167.2 (4)
C7—C6—C8—F4	25.6 (19)	C20—Fe1—C17—C21	37.6 (4)
C5—C6—C8—F4'	−3 (3)	C13—Fe1—C17—C21	−82.9 (5)
C7—C6—C8—F4'	−179 (3)	C12—Fe1—C17—C18	−166.3 (6)
C11—N2—C9—N1	−176.6 (4)	C19—Fe1—C17—C18	−37.5 (5)
C11—N2—C9—S1	−1.0 (7)	C16—Fe1—C17—C18	39.8 (10)
C4—N1—C9—N2	−171.2 (5)	C14—Fe1—C17—C18	115.7 (5)
C4—N1—C9—S1	13.0 (8)	C15—Fe1—C17—C18	73.8 (6)
C9—N2—C11—C12	140.7 (5)	C20—Fe1—C17—C18	−81.5 (5)
C9—N2—C11—C10	−96.2 (6)	C13—Fe1—C17—C18	158.1 (5)
N2—C11—C12—C16	84.9 (5)	C21—Fe1—C17—C18	−119.1 (7)
C10—C11—C12—C16	−35.5 (7)	C21—C17—C18—C19	0.8 (7)
N2—C11—C12—C13	−85.0 (6)	Fe1—C17—C18—C19	60.7 (5)
C10—C11—C12—C13	154.6 (5)	C21—C17—C18—Fe1	−59.9 (4)
N2—C11—C12—Fe1	179.1 (4)	C12—Fe1—C18—C19	46.2 (11)
C10—C11—C12—Fe1	58.6 (6)	C16—Fe1—C18—C19	76.7 (6)
C18—Fe1—C12—C16	40.5 (9)	C14—Fe1—C18—C19	159.6 (5)
C19—Fe1—C12—C16	74.8 (4)	C15—Fe1—C18—C19	117.7 (5)
C14—Fe1—C12—C16	−81.1 (3)	C17—Fe1—C18—C19	−118.1 (7)
C15—Fe1—C12—C16	−37.3 (3)	C20—Fe1—C18—C19	−37.6 (5)
C17—Fe1—C12—C16	−167.0 (7)	C13—Fe1—C18—C19	−170.2 (5)
C20—Fe1—C12—C16	116.9 (4)	C21—Fe1—C18—C19	−80.8 (5)
C13—Fe1—C12—C16	−118.2 (4)	C12—Fe1—C18—C17	164.4 (7)
C21—Fe1—C12—C16	158.9 (4)	C19—Fe1—C18—C17	118.1 (7)
C18—Fe1—C12—C13	158.7 (8)	C16—Fe1—C18—C17	−165.2 (4)
C19—Fe1—C12—C13	−167.0 (4)	C14—Fe1—C18—C17	−82.3 (5)
C16—Fe1—C12—C13	118.2 (4)	C15—Fe1—C18—C17	−124.2 (5)
C14—Fe1—C12—C13	37.1 (3)	C20—Fe1—C18—C17	80.5 (5)
C15—Fe1—C12—C13	80.9 (3)	C13—Fe1—C18—C17	−52.1 (9)
C17—Fe1—C12—C13	−48.8 (8)	C21—Fe1—C18—C17	37.3 (4)
C20—Fe1—C12—C13	−124.9 (4)	C17—C18—C19—C20	−0.4 (8)
C21—Fe1—C12—C13	−82.8 (4)	Fe1—C18—C19—C20	60.4 (5)
C18—Fe1—C12—C11	−79.7 (10)	C17—C18—C19—Fe1	−60.7 (5)
C19—Fe1—C12—C11	−45.4 (6)	C12—Fe1—C19—C18	−162.7 (4)
C16—Fe1—C12—C11	−120.2 (6)	C16—Fe1—C19—C18	−121.1 (5)
C14—Fe1—C12—C11	158.7 (5)	C14—Fe1—C19—C18	−44.2 (9)
C15—Fe1—C12—C11	−157.5 (5)	C15—Fe1—C19—C18	−78.5 (6)
C17—Fe1—C12—C11	72.8 (9)	C17—Fe1—C19—C18	38.8 (5)
C20—Fe1—C12—C11	−3.3 (6)	C20—Fe1—C19—C18	119.6 (7)
C13—Fe1—C12—C11	121.6 (6)	C13—Fe1—C19—C18	166.1 (7)
C21—Fe1—C12—C11	38.7 (6)	C21—Fe1—C19—C18	82.2 (5)
C16—C12—C13—C14	2.5 (5)	C18—Fe1—C19—C20	−119.6 (7)
C11—C12—C13—C14	173.9 (4)	C12—Fe1—C19—C20	77.7 (6)
Fe1—C12—C13—C14	−57.8 (3)	C16—Fe1—C19—C20	119.3 (5)
C16—C12—C13—P1	177.6 (3)	C14—Fe1—C19—C20	−163.8 (5)
C11—C12—C13—P1	−11.0 (7)	C15—Fe1—C19—C20	161.9 (5)
Fe1—C12—C13—P1	117.4 (3)	C17—Fe1—C19—C20	−80.8 (5)
C16—C12—C13—Fe1	60.2 (3)	C13—Fe1—C19—C20	46.5 (11)
C11—C12—C13—Fe1	−128.4 (5)	C21—Fe1—C19—C20	−37.4 (4)
C22—P1—C13—C14	−97.6 (4)	C18—C19—C20—C21	−0.2 (7)
C28—P1—C13—C14	7.6 (5)	Fe1—C19—C20—C21	59.7 (4)
C22—P1—C13—C12	88.2 (4)	C18—C19—C20—Fe1	−59.9 (5)
C28—P1—C13—C12	−166.6 (4)	C18—Fe1—C20—C21	−82.2 (5)
C22—P1—C13—Fe1	174.0 (3)	C12—Fe1—C20—C21	118.5 (4)
C28—P1—C13—Fe1	−80.8 (4)	C19—Fe1—C20—C21	−119.0 (7)
C18—Fe1—C13—C14	−41.9 (7)	C16—Fe1—C20—C21	162.1 (4)
C12—Fe1—C13—C14	120.4 (4)	C14—Fe1—C20—C21	36.5 (11)
C19—Fe1—C13—C14	160.4 (8)	C15—Fe1—C20—C21	−160.0 (6)
C16—Fe1—C13—C14	82.0 (3)	C17—Fe1—C20—C21	−37.7 (4)
C15—Fe1—C13—C14	38.0 (3)	C13—Fe1—C20—C21	76.0 (5)
C17—Fe1—C13—C14	−79.5 (4)	C18—Fe1—C20—C19	36.8 (5)
C20—Fe1—C13—C14	−163.5 (4)	C12—Fe1—C20—C19	−122.4 (5)
C21—Fe1—C13—C14	−122.1 (4)	C16—Fe1—C20—C19	−78.8 (6)
C18—Fe1—C13—C12	−162.3 (6)	C14—Fe1—C20—C19	155.6 (8)
C19—Fe1—C13—C12	40.0 (10)	C15—Fe1—C20—C19	−41.0 (9)
C16—Fe1—C13—C12	−38.4 (3)	C17—Fe1—C20—C19	81.4 (5)
C14—Fe1—C13—C12	−120.4 (4)	C13—Fe1—C20—C19	−164.9 (5)
C15—Fe1—C13—C12	−82.4 (3)	C21—Fe1—C20—C19	119.0 (7)
C17—Fe1—C13—C12	160.2 (4)	C19—C20—C21—C17	0.7 (7)
C20—Fe1—C13—C12	76.1 (4)	Fe1—C20—C21—C17	59.5 (4)
C21—Fe1—C13—C12	117.5 (4)	C19—C20—C21—Fe1	−58.8 (4)
C18—Fe1—C13—P1	79.6 (7)	C18—C17—C21—C20	−0.9 (7)
C12—Fe1—C13—P1	−118.1 (5)	Fe1—C17—C21—C20	−59.7 (4)
C19—Fe1—C13—P1	−78.1 (10)	C18—C17—C21—Fe1	58.8 (4)
C16—Fe1—C13—P1	−156.5 (4)	C18—Fe1—C21—C20	80.7 (5)
C14—Fe1—C13—P1	121.6 (5)	C12—Fe1—C21—C20	−80.7 (5)
C15—Fe1—C13—P1	159.6 (4)	C19—Fe1—C21—C20	37.8 (4)
C17—Fe1—C13—P1	42.1 (5)	C16—Fe1—C21—C20	−42.8 (8)
C20—Fe1—C13—P1	−41.9 (5)	C14—Fe1—C21—C20	−167.6 (4)
C21—Fe1—C13—P1	−0.5 (5)	C15—Fe1—C21—C20	153.9 (7)
C12—C13—C14—C15	−2.3 (5)	C17—Fe1—C21—C20	118.8 (6)
P1—C13—C14—C15	−177.3 (3)	C13—Fe1—C21—C20	−124.6 (4)
Fe1—C13—C14—C15	−59.7 (3)	C18—Fe1—C21—C17	−38.1 (4)
C12—C13—C14—Fe1	57.4 (3)	C12—Fe1—C21—C17	160.5 (4)
P1—C13—C14—Fe1	−117.6 (4)	C19—Fe1—C21—C17	−81.0 (5)
C18—Fe1—C14—C15	−79.2 (4)	C16—Fe1—C21—C17	−161.6 (6)
C12—Fe1—C14—C15	81.5 (3)	C14—Fe1—C21—C17	73.5 (5)
C19—Fe1—C14—C15	−48.4 (7)	C15—Fe1—C21—C17	35.1 (10)
C16—Fe1—C14—C15	37.4 (3)	C20—Fe1—C21—C17	−118.8 (6)
C17—Fe1—C14—C15	−122.1 (4)	C13—Fe1—C21—C17	116.6 (5)
C20—Fe1—C14—C15	169.7 (8)	C13—P1—C22—C23	11.6 (5)
C13—Fe1—C14—C15	118.8 (4)	C28—P1—C22—C23	−91.3 (5)
C21—Fe1—C14—C15	−162.3 (4)	C13—P1—C22—C27	−166.5 (4)
C18—Fe1—C14—C13	162.0 (4)	C28—P1—C22—C27	90.7 (5)
C12—Fe1—C14—C13	−37.3 (3)	C27—C22—C23—C24	0.0 (9)
C19—Fe1—C14—C13	−167.2 (6)	P1—C22—C23—C24	−178.1 (4)
C16—Fe1—C14—C13	−81.4 (3)	C22—C23—C24—C25	0.3 (9)
C15—Fe1—C14—C13	−118.8 (4)	C23—C24—C25—C26	−0.9 (11)
C17—Fe1—C14—C13	119.1 (4)	C24—C25—C26—C27	1.1 (12)
C20—Fe1—C14—C13	50.9 (10)	C25—C26—C27—C22	−0.7 (11)
C21—Fe1—C14—C13	78.9 (4)	C23—C22—C27—C26	0.2 (9)
C13—C14—C15—C16	1.3 (5)	P1—C22—C27—C26	178.4 (5)
Fe1—C14—C15—C16	−58.7 (3)	C13—P1—C28—C33	−80.4 (5)
C13—C14—C15—Fe1	60.0 (3)	C22—P1—C28—C33	23.1 (6)
C18—Fe1—C15—C16	−122.5 (4)	C13—P1—C28—C29	97.9 (5)
C12—Fe1—C15—C16	37.5 (3)	C22—P1—C28—C29	−158.6 (5)
C19—Fe1—C15—C16	−82.2 (4)	C33—C28—C29—C30	2.6 (10)
C14—Fe1—C15—C16	119.8 (4)	P1—C28—C29—C30	−175.7 (5)
C17—Fe1—C15—C16	−163.3 (4)	C28—C29—C30—C31	−1.9 (11)
C20—Fe1—C15—C16	−52.9 (7)	C29—C30—C31—C32	−1.5 (14)
C13—Fe1—C15—C16	81.7 (3)	C30—C31—C32—C33	3.9 (13)
C21—Fe1—C15—C16	170.3 (7)	C29—C28—C33—C32	−0.2 (10)
C18—Fe1—C15—C14	117.7 (4)	P1—C28—C33—C32	178.1 (5)
C12—Fe1—C15—C14	−82.3 (3)	C31—C32—C33—C28	−3.1 (11)
C19—Fe1—C15—C14	158.1 (4)		

### Hydrogen-bond geometry (Å, º)

**Table:**

*D*—H···*A*	*D*—H	H···*A*	*D*···*A*	*D*—H···*A*
N1—H1···S1^i^	0.86	2.46	3.302 (5)	168
N2—H2···S1^i^	0.86	2.64	3.445 (4)	157

Symmetry code: (i) −*y*+3/2, *x*−1/2, *z*−1/4.
